# Characterization of lincRNA expression in the human retinal pigment epithelium and differentiated induced pluripotent stem cells

**DOI:** 10.1371/journal.pone.0183939

**Published:** 2017-08-24

**Authors:** Elizabeth D. Au, Rosario Fernandez-Godino, Tadeusz J. Kaczynksi, Maria E. Sousa, Michael H. Farkas

**Affiliations:** 1 Department of Ophthalmology, Jacobs School of Medicine and Biomedical Science, State University of New York at Buffalo, Buffalo, NY, United States of America; 2 Ocular Genomics Institute, Department of Ophthalmology, Massachusetts Eye and Ear Infirmary, Harvard Medical School, Boston, MA, United States of America; 3 Research Service, Veterans Administration Western New York Healthcare System, Buffalo, NY, United States of America; 4 Department of Biochemistry, Jacobs School of Medicine and Biomedical Science, State University of New York at Buffalo, Buffalo, NY, United States of America; University of Florida, UNITED STATES

## Abstract

Long intervening non-coding RNAs (lincRNAs) are increasingly being implicated as important factors in many aspects of cellular development, function, and disease, but remain poorly understood. In this study, we examine the human retinal pigment epithelium (RPE) lincRNA transcriptome using RNA-Seq data generated from human fetal RPE (fRPE), RPE derived from human induced pluripotent stem cells (iPS-RPE), and undifferentiated iPS (iPS). In addition, we determine the suitability of iPS-RPE, from a transcriptome standpoint, as a model for use in future studies of lincRNA structure and function. A comparison of gene and isoform expression across the whole transcriptome shows only minimal differences between all sample types, though fRPE and iPS-RPE show higher concordance than either shows with iPS. Notably, RPE signature genes show the highest degree of fRPE to iPS-RPE concordance, indicating that iPS-RPE cells provide a suitable model for use in future studies. An analysis of lincRNAs demonstrates high concordance between fRPE and iPS-RPE, but low concordance between either RPE and iPS. While most lincRNAs are expressed at low levels (RPKM < 10), there is a high degree of concordance among replicates within each sample type, suggesting the expression is consistent, even at levels subject to high variability. Finally, we identified and annotated 180 putative novel genes in the fRPE samples, a majority of which are also expressed in the iPS-RPE. Overall, this study represents the first characterization of lincRNA expression in the human RPE, and provides a model for studying the role lincRNAs play in RPE development, function, and disease.

## Introduction

The retinal pigment epithelium (RPE), a single cell layer in the posterior eye, is integral for maintaining visual function [1]. While not directly involved in light perception, it closely interacts with rod and cone photoreceptors, and serves a multitude of functions, acting as a gateway between the retina and the rest of body. As a polarized cell, the RPE has long apical microvilli that interdigitate with photoreceptor outer segments, allowing for the exchange of nutrients, ion transport, and phagocytosis. Additionally, melanin is the main pigment of the RPE, and is responsible for light absorption, helping to protect the eye from light-induced damage. The RPE has been shown to be a key cell type where disease pathogenesis begins, and loss of any one of its functions can cause retinal degeneration, and, ultimately, vision loss [2–6].

A myriad of diseases affect RPE function, including inherited retinal dystrophies (IRDs), diabetes, and macular degeneration [7–9]. To address the complexity of issues relating to loss of function and disease pathogenesis, vision research is focused in 3 integrated areas: 1) identifying mutations, or other factors, that cause vision loss, 2) understanding the molecular mechanisms underlying disease pathogenesis, and 3) discovering therapies that can preserve sight, such as gene therapy [10–15]. There are many factors that need to come together in order to realize the full potential of these research initiatives. For example, to identify disease-causing mutations, thorough genomic annotations and well characterized gene expression are critical [16]. Further, studying the functional effects of, and developing a therapeutic strategy to combat the effects of, these mutations requires an appropriate model. The RPE is a known site of disease pathogenesis, and it can be readily differentiated from induced pluripotent stem (iPS) cells, so it is an ideal cell type for both gene expression and functional studies, as well as for therapeutic studies [17–19].

While the human RPE transcriptome has been characterized using both microarray and RNA-Seq methods, genomic annotation databases are still lacking, and the tissue- and cell-specific nature of gene expression adds a new layer of complexity to defining a baseline for analysis [20,21]. The wealth of information contained in high throughput sequencing data provides an opportunity to better annotate the genome and define tissue- and cell-specific gene expression profiles, including the addition of information about long intervening non-coding RNAs (lincRNAs) [16,22,23]. lincRNAs have so far been largely unstudied. While research into lincRNAs is increasing rapidly, and many more are being identified, our understanding of them is still minimal [24]. At the transcript level, lincRNAs are like their protein-coding counterparts. They are transcribed by RNA polymerase II, have a 5’ cap and 3’ poly-A tail, are multi-exonic, and at least 25% are alternatively spliced [24,25]. Roughly 21,650 lincRNA genes have been annotated, accounting for 22,518 isoforms [22]. Importantly, they are increasingly being associated with disease. In fact, lincRNAs have been identified to cause or regulate the severity of pathogenesis for a variety of cancers and Alzheimer’s, and have also been shown to be associated with visual dystrophies [26–30].

Finding an appropriate model to study potential disease-related genetic mutations, including those in lincRNAs, is challenging for vision research. Mouse models are predominantly used to study visual systems, but are not always appropriate, since mice do not completely share structural features of the eye, such as the macula, with humans, and gene expression and alternative splicing are not well conserved [31–33]. With the advent of iPS cell technologies, building disease models directly from affected patients or with CRISPR/Cas9 genome editing techniques is becoming more common, allowing for the creation of a more suitable model [34–36]. For example, previous studies used iPS-RPE to model the retinal dystrophies Best vitelliform macular dystrophy and age-related macular degeneration [37]. This is providing a more streamlined pipeline for determining the mechanism of disease pathogenesis, which should aid the development of therapeutic strategies, such as gene therapy or cell replacement therapy [38–40].

The present study was designed to address both the lack of knowledge about lincRNA gene expression and the need to identify a suitable model to study lincRNAs in vision research, particularly in the RPE. Given their importance as transcriptional regulators, potential disease implications, and lack of study in the eye, a thorough analysis of lincRNA expression in the RPE is needed. We used both fetal RPE (fRPE) and RPE derived from iPS (iPS-RPE) in order to determine the suitability of iPS-RPE for use as a model system to study lincRNA function in this important retinal cell type. We identified over 1,000 lincRNAs expressed in the RPE. Importantly, we found that iPS-RPE and fRPE show similar expression profiles for lincRNAs, and are much more highly correlated with each other than either is with the iPS samples. Interestingly, the fRPE and iPS-RPE showed the highest concordance among RPE signature genes, and neither is highly correlated with undifferentiated iPS cells. We also identified 180 putative novel genes in the fRPE, which show similar concordance patterns. These data provide the first insights into lincRNA expression in the human RPE, serve as a foundation to study their functional role in health and disease, and show that iPS-RPE are a suitable model for such studies.

## Materials and methods

### Sample acquisition

Human iPS cells (1016 line) were generously provided by Dr. Chad Cowan at the Harvard Stem Cell Institute, from which we differentiated the iPS-RPE line. Human fetal eyes were purchased from Advanced Biomedical Resources, Inc (Alameda, California). All samples for this study were provided to the authors anonymously, thus we did not obtain consent.

### Maintenance and collection of iPS cells

Human iPS cells were seeded at 500,000 cells in a 10-cm dish coated with Matrigel (Corning). Cells were maintained in mTeSR1 media (Stem Cell Technologies) with Rock Inhibitor (Y-27632 dihydrochloride, Santa Cruz Biotechnology) at a final concentration of 1 μM/mL. Media without Rock Inhibitor was changed daily. To obtain 3 technical replicates, we plated and collected cells from a total of 3 individual plates.

### Differentiation of human iPS cells

All reagents were purchased from Invitrogen (Carlsbad, CA) unless noted otherwise. The procedure for differentiating human iPS cells toward RPE was performed as previously described [17]. Briefly, when the iPS culture reached 60–70% confluency, individual colonies were lifted using accutase (Stem Cell Technologies). Colonies were allowed to settle in a 15 mL conical tube (BD), and, after media was replaced with mTeSR 1 media, were transferred to a T25 flask (Corning) to initiate differentiation (day 0). Over the following 4 days, the colonies were gradually transitioned to neural induction medium (NIM) which consisted of DMEM/F12, 1% N2 supplement, MEM non-essential amino acids, and 2 μg/mL heparin. At day 6, the colonies were transferred to a 10-cm dish coated with laminin in NIM, changed every 2 days. Rosettes were removed from the culture by light trituration at day 16. The remaining cells were switched to retinal differentiation medium (DMEM/F12 (3:1), 2% B27 without retinoic acid, and 1% antibiotic/antimycotic). This culture was maintained in RDM until day 80 for dissection and passaging, which was performed as described. This process was performed 3 times to generate the 3 technical replicates used for this study.

### Fetal RPE isolation

Whole fetal eyes from 3 individual fetuses were shipped overnight on wet ice. The eye was dissected just below the ora serata to remove the cornea and lens. The neural retina was removed by incubation with 2 mg/ml Dispase I at 37°C for 1 hour. Following incubation, the neural retina was gently removed The RPE was isolated by gently peeling sheets from the choroid. The RPE was then flash frozen and stored at -80°C prior to RNA isolation.

### RNA isolation, library preparation, and sequencing

Total RNA was extracted from individual RPE samples using the RNeasy Plus Mini kit (Qiagen), according to manufacturer’s instructions. RNA quantity and quality was determined using an RNA Nano 6000 kit (Agilent) on an Agilent BioAnalyzer 2100. RNA libraries were prepared from samples with a RIN > 9.0 using a SureSelect Strand-Specific RNA Library Prep kit (Agilent), according to the manufacturer’s protocol. Libraries were prepared using 100 ng of total RNA and each sample was uniquely indexed for multiplexing. Library sample quantity and quality were determined using the DNA High Sensitivity kit (Agilent) on an Agilent Bioanalyzer 2100. Six samples were multiplexed and clustered at 8 pM in two lanes of a flow cell. 101 bp paired-end sequencing was performed on an Illumina HiSeq 2500.

### Alignment and feature quantification

Reads were aligned to the human genome version hg38 analysis set, which does not include alternate contigs, using STAR (v2.5.2b). A non-redundant transcriptome database was derived from Gencode v24 and lincRNA annotation databases available on the UCSC Genome Browser [41]. Raw counts for each exon were generated using RSubread (v1.24.1), and were summed over each isoform [42]. Only reads uniquely mapping to one genomic location were included in the counts. Uniquely mapped reads falling within overlapping isoforms were counted as belonging to both isoforms, unless a splice junction unique to one particular isoform of a gene had zero reads, in which case that isoform was considered not to be expressed and given a zero count. Splice junctions were quantified using the output provided by the STAR alignment.

### Identification of specific isoforms and downstream analyses

RPKM values were calculated from the raw counts using total reads and transcript length calculated by Rsubread. An isoform was considered expressed if the average RPKM across all samples in a given condition was greater than 1. Specific isoforms were identified by developing a list of unique splice junctions for each isoform in our annotation database (S1 Table). If the splice junction had reads crossing it, we considered that isoform to be expressed. Concordance was calculated using a Pearson correlation in R.

### Identification and annotation of putative novel genes

Novel genes were identified from the whole transcriptome data as previously described [16]. Briefly, novel splice junctions identified by STAR were parsed to identify those that were intergenic based on our annotation database. Splice junctions were then informatically grouped based on their genomic location. Splice junctions that were within 2 kb of each other were grouped as potential candidates of belonging to the same transcript. These regions were flagged, then hand annotated following the splice junctions with the highest read depth. Terminal exons were arbitrarily annotated as 500 bp in length.

## Results

### Whole transcriptome profiles

We generated transcriptomes from 3 technical replicates each of human iPS cells and 3 technical replicates of iPS-RPE derived from the afformentioned iPS cells, as well as 3 biological replicates of 16-week old fRPE using a stranded mRNA-Seq library preparation protocol. The libraries were sequenced on an Illumina HiSeq2500, generating between 56–137 million reads per sample. We first characterized overall gene expression in order to determine the quality of the sample preparations. Over 85% of reads from each sample were able to align uniquely to the hg38 human genome build. Counts were generated using a non-redundant annotation database generated using the GENCODE v24 and lincRNA annotations, which consists of 65,439 genes and 113,246 isoforms [41]. From our annotation database, we created a list of isoform-specific splice junctions, which we used as a reference to determine expression of unique isoforms for a given gene (S1 Table). Using a conservative approach, we considered an isoform to be expressed if it had an RPKM greater than 1 [43]. This method identified 33,689, 34,288, and 35,811 isoforms, representing 14,675, 14,912, and 15,687 unique genes, expressed in the iPS, iPS-RPE, and fRPE samples, respectively (Table 1). Intra-sample gene concordance (i.e.–among the replicates within a condition) was greater than 0.98 for iPS and iPS-RPE samples, and 0.78 for fRPE samples. Relative to the intra-sample gene concordance, intra-sample isoform concordance was lower in the iPS and iPS-RPE samples, but still greater than 0.95. Conversely, the fRPE samples were more concordant at the isoform level, at 0.82. This is an important finding, and suggests that isoform specificity may play in important role in RPE gene expression. This highlights the need to study expression at the isoform rather than the gene level.

**Table 1:** Whole transcriptome intra-sample gene expression and concordance.

*Sample*	*Genes*	*Concordance*	*Isoforms*	*Concordance*
*Fetal RPE (fRPE)*	15,687	0.78	35,811	0.82
*iPS-RPE*	14,912	0.98	34,288	0.95
*iPS*	14,675	0.99	33,689	0.96

**Fig 1:**
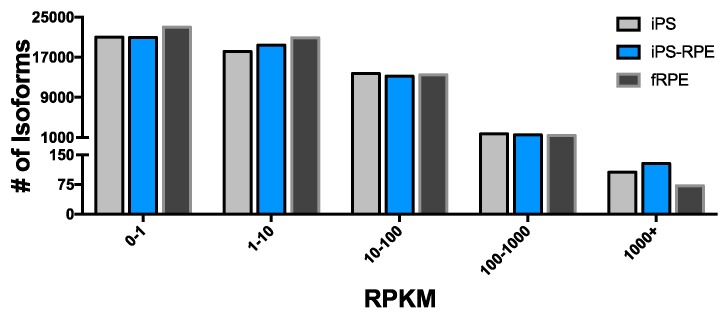
Distribution of transcriptome gene expression. Total number of isoforms with RPKM in each of 5 bins is shown for iPS, iPS-RPE, and fRPE. Isoforms with RPKM greater than 1 are considered to be expressed. The majority of expressed isoforms have RPKM less than 100 for all samples, with very few isoforms having RPKM greater than 1000.

To further assess transcriptome isoform expression, we looked at the RPKM distribution across all isoforms using 6 bins: zero, 0–1, 1–10, 10–100, 100–1000, and greater than 1000. As expected, a majority of isoforms had an RPKM less than 1 and were considered not expressed, based on our conservative definition. (Fig 1). Of the expressed isoforms, the fRPE (20,892) and iPS-RPE (19,426) had more low expressing isoforms (RPKM = 1–10) than the iPS (18129) samples. These represent between 54% (iPS) and 58% (fRPE) of the total expressed isoforms. As would be expected, the iPS then have more medium (RPKM 10–100) and high (RPKM 100–1000) expressing isoforms (13,736/1,718, respectively), while the fRPE (13,473/1,374) and iPS-RPE (13,229/1,505) have fewer in these categories. Overall, the medium to high expressing isoforms represent 46%, 42%, and 41% of the total expressed in iPS, iPS-RPE, and fRPE, respectively.

In order to examine differences between conditions, and begin to determine the suitability of iPS-RPE as a model for RPE studies, we next compared the overlap of isoform expression among the three sample types. We found the highest degree of isoform overlap between the iPS-RPE and fRPE (31,058), with both iPS-RPE and fRPE showing a lesser degree of overlap with iPS (28,791 and 28,850, respectively) (Table 2). Further, a Pearson correlation showed the highest concordance between fRPE and iPS-RPE (0.84), while the iPS to iPS-RPE (0.74) was next highest, and the fRPE to iPS (0.66) was the lowest. The inter-sample concordance shows that not only do fRPE and iPS-RPE have the most overlap in number of expressed isoforms, but expression patterns are also tightly correlated. In addition, the isoform concordance between the fRPE and iPS-RPE matches the gene concordance. The concordance of either RPE with the iPS cells is marginally lower.

**Table 2:** Whole transcriptome inter-sample gene expression and concordance.

*Sample*	*Genes*	*Concordance*	*Isoforms*	*Concordance*
*fRPE:iPS-RPE*	13,481	0.84	31,058	0.84
*fRPE:iPS*	12,425	0.71	28,850	0.66
*iPS:iPS-RPE*	12,470	0.76	28,791	0.74

### RPE signature gene expression

The RPE transcriptome has been characterized by multiple groups, under varying conditions [44–47]. Each study has developed an RPE signature gene list defined by protein-coding genes whose expression is specific to, or enriched in, the RPE. These lists have emerged as useful guides for characterizing RPE differentiated from iPS cells. To further assess the potential for iPS-RPE to be used as a model for the RPE, we examined the expression of the RPE signature genes in these cells, in comparison to both fRPE and iPS.

**Fig 2:**
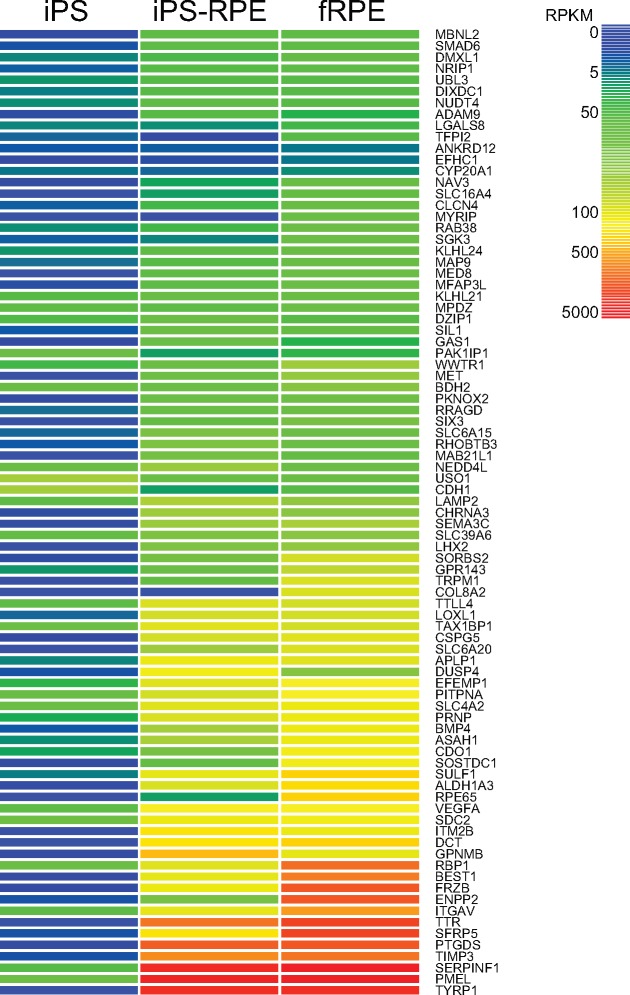
Heatmap of RPE signature genes. Heatmap showing expression levels, in RPKM, of RPE signature genes in iPS, iPS-RPE, and fRPE. Genes are clustered by expression pattern across all three conditions. High expression levels are seen in fPRE and iPS-RPE, with little to no expression in iPS.

We used the RPE signature gene list developed by Liao, et. al (2010), consisting of 86 genes with 292 isoforms [47]. We found that 62, 85, and 86 of these genes were expressed in iPS, iPS-RPE, and fRPE samples, respectively (Table 3). Interestingly, while nearly all RPE signature genes were expressed in fRPE and iPS-RPE, not all isoforms were expressed. The fRPE samples had 245 isoforms expressed, and the iPS-RPE 235 isoforms (Table 3). All but one of the RPE signature isoforms expressed in the iPS-RPE were also expressed in the fRPE. We also compared relative expression levels of the RPE signature genes across the 3 samples. The iPS data show that the RPE genes that are expressed, are expressed at very low levels. A heat map of the RPE genes demonstrates that the iPS-RPE and fRPE show much higher levels of expression, comparatively, though, for the most part, expression is higher in the fRPE than the iPS-RPE (Fig 2). Still, a Pearson correlation shows that fRPE and iPS-RPE samples are very tightly correlated (0.91), while both the fRPE and iPS-RPE have low concordance with the iPS (0.03 and 0.02, respectively) (Table 3). This further confirms that, although absolute expression levels may be different, the fRPE and iPS-RPE show highly similar overall expression patterns for the RPE genes, while those RPE genes expressed in the iPS exhibit different expression patterns as compared to the RPE sample. Unlike the whole transcriptome data, isoform concordance increases, relative to gene concordance, for all comparisons (Table 3, Inter-Sample)

**Table 3:** RPE signature gene expression and concordance.

***Intra-Sample***	***Genes***	***Concordance***	***Isoforms***	***Concordance***
*Fetal RPE (fRPE)*	86	0.93	245	0.93
*iPS-RPE*	85	0.98	235	0.97
*iPS*	62	0.99	164	0.98
***Inter-Sample***	*Genes*	*Concordance*	*Isoforms*	*Concordance*
*fRPE:iPS-RPE*	85	0.91	234	0.93
*fRPE:iPS*	62	0.03	163	0.16
*iPS:iPS-RPE*	61	0.02	160	0.19

### Defining expression of annotated lincRNA in the RPE

Expression of lincRNAs has not been studied nearly as well as the protein-coding transcriptome. In fact, the lincRNA transcriptome has not yet been fully characterized in any human retinal tissue. In this study, we found that 1,156, 1,144, and 1,603 of the 21,650 annotated lincRNA genes are expressed in the iPS, iPS-RPE, and fRPE transcriptome (Table 4). Similar to the intra-sample concordance of the whole transcriptome, the iPS and iPS-RPE had Pearson correlation coefficients over 0.95. The fRPE, however, had lower concordance at 0.73. Relative to the whole transcriptome analysis, far fewer lincRNA isoforms per gene were expressed. Of the 22,518 isoforms, the fewest were expressed in the iPS-RPE (1187), whereas the iPS (1210) and fRPE (1674) had marginally more expressed. Not surprisingly, this small number of isoforms expressed per gene resulted in no change in intra-sample concordance when we look at the gene versus the isoform level.

**Table 4:** lincRNA intra-sample gene expression and concordance.

*Sample*	*Genes*	*Concordance*	*Isoforms*	*Concordance*
*Fetal RPE (fRPE)*	1,603	0.73	1,674	0.73
*iPS-RPE*	1,144	0.95	1,187	0.95
*iPS*	1,156	0.95	1,210	0.95

**Fig 3:**
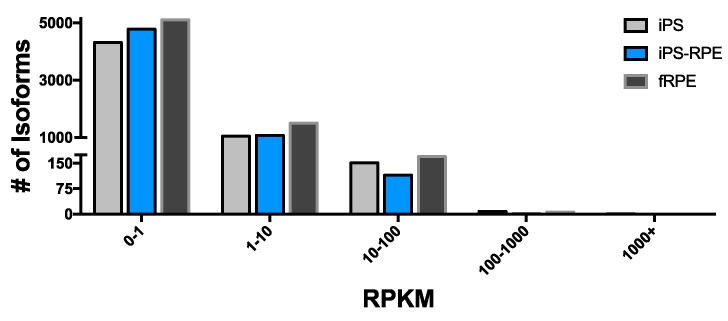
lincRNA expression distribution. Total number of lincRNA isoforms with RPKM in each of 5 bins in shown for iPS, iPS-RPE, and fRPE. lincRNAs with RPKM greater than 1 are considered to be expressed. The majority of expressed lincRNAs have RPKM less than 10, with very few having RPKM above 100.

Previous studies have shown that lincRNAs are typically expressed at low levels [25,48], and we find this to be true in our study as well. The vast majority of expressed lincRNAs have RPKM levels below 10 for all three conditions: 1,050 in iPS, 1,071 in iPS-RPE, and 1,498 in fRPE (Fig 3). These represent 86–90% of the expressed lincRNAs. We find just over 100 lincRNAs with RPKM above 10 in each condition, representing 10–12% of expressed lincRNAs. Very few lincRNAs were detected with RPKM above 100: 9 in iPS, 6 in fRPE, and 1 in iPS-RPE.

**Fig 4:**
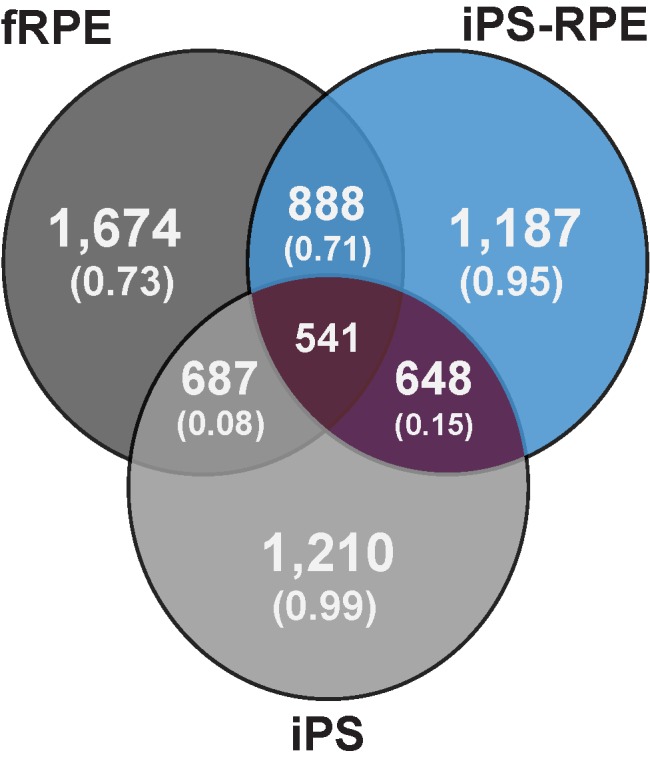
lincRNA expression and concordance. Outer regions show total number of lincRNAs expressed in each sample, with the intra-sample concordance in parentheses below. Overlapping regions contain total numbers of lincRNAs expressed in both sample types, with the inter-sample concordance in parentheses below. Center region contains the total number of lincRNAs expressed in all three sample types.

Overlap of lincRNA isoforms expressed between the three samples showed an interesting trend. Seventy four percent of lincRNA isoforms expressed in iPS-RPE are also expressed in fRPE (888), while only just over 50% of lincRNA isoforms expressed in iPS were expressed in iPS-RPE and fRPE (648 and 687, respectively) (Fig 4). A Pearson correlation better exemplifies the comparison of lincRNA expression, where fRPE and iPS-RPE show a correlation of 0.71, while fRPE and iPS-RPE show considerably lower correlation with iPS (0.08 and 0.15, respectively). Importantly, not only do we find fewer lincRNAs expressed in both iPS and either RPE sample, those that are overlapping do not exhibit concordant expression patterns, while the lincRNAs expressed in both RPE samples indeed show similar patterns of expression.

### Analysis of cellular contamination in fRPE samples

An important consideration for transcriptome studies of the RPE derived from whole eyes is contamination from surrounding cell types, such as photoreceptors and choroid [49].To determine the extent to which our fRPE samples were contaminated with these other cell types, we looked at expression of protein-coding genes that are expressed more highly in choroid and photoreceptors relative to RPE. We used the table generated by Bennis, et al., (2015) which consisted of 583 genes more highly expressed in choroid and 1582 genes more highly expressed in photoreceptors [49]. Of these, 482 choroid genes and 1177 photoreceptor genes were in our reference transcriptome database. Since RNA-Seq has the power to differentiate and quantitate isoforms, we expanded the gene list to include 1532 and 3701 isoforms for the choroid and photoreceptor genes, respectively. Based on the conclusions of Bennis, et al. (2015), we would expect the expression of these contaminating genes to be skewed toward higher expression levels in the fRPE in cases where significant contamination is present, while the iPS and iPS-RPE will skew toward low-to-no expression. In fact, when we binned the contaminating genes we found roughly equal numbers across the bins for each sample type (S1 Fig). A majority of the isoforms were found to be not expressed (RPKM < 1) in any of the sample types. Interestingly, of those that were expressed, the iPS and iPS-RPE samples had more genes expressed at medium (RPKM = 10–100) to high levels (RPKM = 100–1000+), than the fRPE samples. Conversely, the fRPE samples did have marginally more isoforms expressed at low levels (RPKM 1–10) than either iPS or iPS-RPE.

### Annotation and characterization of novel genes

**Fig 5:**
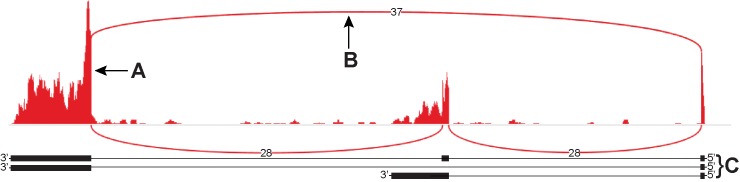
Sashimi plot for a novel gene detected in the fRPE. **A)** Coverage plot showing alignment of reads to the genome. **B)** Curved lines represent splice junctions. The number in the line represents the number of reads crossing a particular splice junction. **C)** Predicted annotation for 3 isoforms of this novel gene based on coverage and splice junction data.

Our analysis of the fRPE transcriptome identified a set of novel splice junctions that lie outside annotated regions of the genome, allowing us to define these as putative novel genes, highlighting the current lack of complete genome annotation. We manually annotated these novel genes and identified 180 that were at least 3 exons in length, with an example shown in Fig 5. We also detected over 450 novel genes with 2 exons, but did not annotate these because we cannot properly annotate terminal exons with RNA-Seq data; this requires empiric analysis [16,25]. Among the 180 novel genes we analyzed, the number of exons range from 3 to 9, with an average of 3.7 exons per gene. Over half (111) had more than one expressed isoform. The average internal exon length was 152 bp (minimum length = 18 bp, and maximum length = 1262 bp), and the average transcript length was 278 bp, excluding terminal exons. These novel genes varied in expression level with an average RPKM of 0.5, and the highest expressing novel gene having an RPKM of 29. Because expression levels were so low, we did not use a minimum RPKM requirement for novel genes to be considered expressed. Of the 180 novel genes, we detected 156 in at least one iPS-RPE sample, and 78 in at least one iPS sample. The intra-sample concordance of the novel genes followed similar trends as was seen for annotated genes: 0.79 in the fRPE, 0.98 in the iPS-RPE, and 0.93 in the iPS. Similarly, we saw inter-sample concordance trends that resembled those seen for annotated genes, with fRPE and iPS-RPE showing the highest concordance at 0.49, while iPS and iPS-RPE as well as fRPE and iPS concordance were much lower at 0.19 and -0.003, respectively. We have included a GTF file of the novel genes in S2 Table.

## Discussion

The goal of this study was two-fold: to characterize lincRNA expression in the human fetal RPE, and to determine if iPS-RPE can serve as a suitable model for future studies of the role lincRNAs play in RPE function. We designed our studies specifically to accomplish these goals. Namely, we used fetal RPE freshly isolated from the eye to get the best picture of *in vivo* lincRNA expression. Second, we compared iPS and iPS-RPE derived from the same iPS line to determine the degree of differentiation of the RPE from its parent cell line.

The ENCODE project has served as an important resource for the study of lincRNAs in many tissues and systems, but the data are lacking in the visual system, since the eye was not included in the initial project [50,51]. To that end, we have previously used RNA-Seq to identify novel lincRNAs expressed in the human neural retina [16]. However, since the neural retina consists of over 60 cell types, and it is known that gene expression is at least partly cell-specific, this set of data is limited in its applicability [52,53]. In addition, lincRNA expression is likely somewhat species-specific [25,54]. Mustafi et al., (2013) reported on the sequence conservation of lincRNAs expressed in the mouse eye to lincRNAs expressed in the human eye [55]. The authors identified 18 conserved lincRNAs, but given that we identified over 1000 lincRNAs to be expressed in the RPE alone, this dataset must be expanded.

We chose to characterize lincRNA expression in human RPE because it is a vital cell type for maintaining retinal function, it is an important site of disease pathogenesis, it can be readily differentiated from iPS cells, and it has a defined set of functions that can be assayed *in vitro* [1,56]. Before functional studies of lincRNAs in the RPE can begin, it is important to identify the lincRNA genes, and, in particular, the isoforms, expressed in the RPE. We used human fetal RPE as the gold-standard for gene expression in the RPE, to which we could compare iPS-RPE gene expression. We specifically chose to analyze RPE freshly isolated from fetal eyes, rather than cultured fRPE, because our goal is to define the human RPE lincRNA transcriptome as naturally as possible. Culturing fRPE would likely change the lincRNA transcriptome, potentially introducing artefacts into our data, or altering the levels of expressed genes [57]. On a whole transcriptome level, other studies have shown that, while iPS-RPE are similar to fRPE, they are less so than RPE derived from human embryonic stem cells [46]. While we cannot draw a comparison to embryonic stem cells, it is true that, when we examined the whole transcriptome, we observed only a marginal increase in Pearson correlation between fRPE and iPS-RPE as compared to that of the iPS and iPS-RPE. This is not too surprising, however, since the fRPE were obtained from 3 individuals, so we would expect slight differences in the expression profile, whereas the iPS-RPE were derived from the undifferentiated iPS. Additionally, the iPS were reprogrammed from fibroblasts, and the epigenetic landscape is believed to change, minimally, during reprogramming and differentiation [58,59]. This suggests that a certain degree of gene expression is similar to the parent cell type, resulting in the observed similarities between the iPS and iPS-RPE as well as the differences between the fRPE and iPS-RPE at the whole transcriptome level.

Importantly, from the perspective of the RPE transcriptome, our analyses demonstrate that the fRPE and iPS-RPE are extremely similar. Of the 86 RPE signature genes, the iPS-RPE expressed 85, and fRPE expressed all 86. As the heat map in Fig 2 suggests, though the genes are expressed at slightly higher levels in the fRPE, the overall pattern of expression is highly correlated. This is confirmed by the higher Pearson correlation between the fRPE and iPS-RPE as compared to the full transcriptome correlation. In addition, the expression profile of these RPE signature genes in fRPE and iPS-RPE are quite different from the undifferentiated iPS samples. Two points can be inferred from these findings. First, the iPS-RPE, at a minimum, have the requisite genes being expressed to perform the multitude of RPE functions, and their expression closely mimics that seen in fRPE. Second, the iPS-RPE are much less concordant at the RPE signature gene level with the iPS cells, a change that could not be observed at the whole transcriptome level. This highlights the fact that iPS-RPE cells are, in fact, more similar to fRPE cells than to their parent iPS cells with respect to genes that are typically used to define the RPE.

Transcriptome analysis of lincRNAs is considered more difficult than that of protein-coding genes because lincRNA expression is notably low [22]. Analysis of gene expression in the present study suggests that nearly all expressed lincRNAs fall within the 1–10 RPKM range, and the rest in the 10–100 RPKM range. Lower expressing genes are, in general, more variable in RNA-Seq data [60,61]. These observations would suggest that the lower expressing lincRNAs would have greater variability, and likely be less concordant between both replicate samples, and, almost certainly, across sample types. While we do see this to a certain extent, the change in concordance is not consistent, leading us to believe it is not driven solely by lower expression levels. The intra-sample concordance is lower, but only marginally. The concordance between sample types demonstrates that, at the lincRNA level, the iPS-RPE and fRPE are the most alike, and we see extremely low concordance between the RPE samples and the iPS cells. Further, the concordance between RPE samples and iPS is much lower for lincRNAs than is seen in the full transcriptome, while the fRPE to iPS-RPE concordance is still relatively high. This speaks to the notion that lincRNA expression is tissue- or cell- type specific, to a much greater degree than is seen transcriptome wide. For future studies of lincRNA function, and its role in RPE homeostasis and function, this is an important finding. Additionally, the higher concordance between the iPS-RPE with the fRPE suggests that the overlap of lincRNA expression is, in fact, real and not an artefact. One could hypothesize that this relatively tight regulation of lincRNA expression is important for RPE differentiation, development, and/or maintenance. Certainly, this will require extensive studies to corroborate. While it is too early to make generalizations about the role the expressed lincRNAs may play in the RPE, since the function for a vast majority of the lincRNAs is unknown, this finding suggests, at the very least, that iPS-RPE would indeed be a useful model for performing studies of lincRNAs in the RPE.

It is difficult to fully remove contaminating cell types when isolating RPE from whole eyes for transcriptome studies. Furthermore, it is difficult to fully quantify the amount of gene expression attributable to contaminating tissues since so few truly tissue-specific genes have been identified. The best method available is to use sets of genes that are found to be more highly expressed in one cell type relative to another. To examine the level of contamination present in our samples, we used data generated by Bennis, et al. (2015) that contained separate sets of genes more highly expressed in choroid and photoreceptors relative to RPE. We found that a majority of these genes are not expressed in any of the sample types. This is potentially attributed to the fact that the gene sets were generated in mouse RPE using microarray technology, whereas our study uses RNA-Seq to explore the human RPE transcriptome. Regardless, we still identified hundreds to thousands of isoforms from these datasets expressed in our samples. Since these genes are supposed to be expressed more highly in choroid and/or photoreceptors, we would expect fRPE to contain more genes that are expressed at higher levels than either the iPS or iPS-RPE samples, in cases where significant contamination is present. In fact, we observed the opposite to be true. This suggests that while the fRPE surely contains data from contaminating cell types, it is minimal and unlikely to affect the results. Furthermore, particularly for lincRNAs, it will be imperative for future studies to independently confirm expression and sub-cellular localization in the tissue of interest prior to undertaking large-scale studies.

Finally, we have identified close to 200 putative novel genes in the human fetal RPE. As with our earlier reporting on the detection of novel genes in the human neural retina, we stayed conservative with our definition of a gene [16]. In particular, to be considered a novel gene, we must have seen at least 3 intergenic exons. These novel genes typically consist of short exons, and, as noted, have very low levels of expression. It is not surprising, therefore, that these putative genes have been missed in other transcriptome analyses. Of the 180 putative novel genes, 156 were also seen in the iPS-RPE, while 78 were observed in the iPS cells. This would suggest that they are not all RPE-specific, though a majority may indeed be. Since it is not possible to detect the true transcription start and stop sites using RNA-Seq data alone, we chose to arbitrarily assign each terminal exon to be 500 bp in length. In most cases, this will likely be an over-estimation, and it will be important for future studies of these novel genes to empirically determine the length of the terminal exons.

## Conclusion

In this study, we characterized lincRNA expression in two sources of RPE: fetal RPE and RPE derived from iPS cells. We found a high degree of similarity between the two, both in terms of overall lincRNA expression and pattern of expression. Further, we found very little similarity in lincRNA expression patterns between either RPE and undifferentiated iPS cells. This exemplifies the notion that lincRNA expression is tissue- and cell- type specific. In addition, we have demonstrated that the iPS-RPE are a suitable model to study lincRNA function. The highest concordance seen throughout all our analyses was found when comparing the fRPE and iPS-RPE samples for RPE signature gene expression, leading us to conclude that the iPS-RPE cells provide a good model for RPE studies. Since human RPE is extremely difficult to procure, and current models are lacking, this is an important result, and is extremely promising to promote future studies examining RPE gene expression, structure, and function, and testing potential disease treatments.

## Supporting information

S1 Table Table of unique lincRNA splice junctions. (TXT)

S2 Table Table of novel genes. (TXT)

S1 Fig Graph of gene expression profiles for highly expressed photoreceptor and choroid genes relative to RPE. (EPS)
